# Neuroanatomical Study of the A11 Diencephalospinal Pathway in the Non-Human Primate

**DOI:** 10.1371/journal.pone.0013306

**Published:** 2010-10-13

**Authors:** Quentin Barraud, Ibrahim Obeid, Incarnation Aubert, Gregory Barrière, Hugues Contamin, Steve McGuire, Paula Ravenscroft, Gregory Porras, François Tison, Erwan Bezard, Imad Ghorayeb

**Affiliations:** 1 Université Victor Segalen Bordeaux 2, Centre National de la Recherche Scientifique, Bordeaux Institute of Neuroscience, UMR 5227, Bordeaux, France; 2 Centre Hospitalier et Universitaire de Bordeaux, Bordeaux, France; 3 Cynbiose, Marcy l'Etoile, France; 4 Motac Neuroscience Ltd, Manchester, United Kingdom; Emory University, United States of America

## Abstract

**Background:**

The A11 diencephalospinal pathway is crucial for sensorimotor integration and pain control at the spinal cord level. When disrupted, it is thought to be involved in numerous painful conditions such as restless legs syndrome and migraine. Its anatomical organization, however, remains largely unknown in the non-human primate (NHP). We therefore characterized the anatomy of this pathway in the NHP.

**Methods and Findings:**

In situ hybridization of spinal dopamine receptors showed that D1 receptor mRNA is absent while D2 and D5 receptor mRNAs are mainly expressed in the dorsal horn and D3 receptor mRNA in both the dorsal and ventral horns. Unilateral injections of the retrograde tracer Fluoro-Gold (FG) into the cervical spinal enlargement labeled A11 hypothalamic neurons quasi-exclusively among dopamine areas. Detailed immunohistochemical analysis suggested that these FG-labeled A11 neurons are tyrosine hydroxylase-positive but dopa-decarboxylase and dopamine transporter-negative, suggestive of a L-DOPAergic nucleus. Stereological cell count of A11 neurons revealed that this group is composed by 4002±501 neurons per side. A 1-methyl-4-phenyl-1, 2, 3, 6-tetrahydropyridine (MPTP) intoxication with subsequent development of a parkinsonian syndrome produced a 50% neuronal cell loss in the A11 group.

**Conclusion:**

The diencephalic A11 area could be the major source of L-DOPA in the NHP spinal cord, where it may play a role in the modulation of sensorimotor integration through D2 and D3 receptors either directly or indirectly via dopamine formation in spinal dopa-decarboxylase-positives cells.

## Introduction

A number of pathological conditions are related to dysregulation of dopaminergic transmission such as schizophrenia and the mesocortical system, addiction and the mesolimbic system, and Parkinson's disease (PD) and the nigrostriatal system [1], [2], [3]. Contrasting with the forebrain, the dopaminergic innervation of the spinal cord from A11 remains largely under-investigated [4], [5], [6] although its functional implications in the spinal cord autonomic and sensory-motor processes are of particular relevance for pain control [7], cataplexy [8], locomotor network modulation [9] and painful human conditions such as restless legs syndrome (RLS) [10] and migraine [11]). For example, spinal dopamine (DA) contributes to spinal reflex excitability where it depresses monosynaptic “stretch” reflexes via D2 and D3 receptors [12], [13]. In rodents, it has been suggested that DA release may reduce the behavioral responses to noxious stimulation [7], [14] and may significantly inhibit the nociceptive processes in the trigeminocervical complex [11]. DA turnover in the spinal dorsal horn increases in response to noxious stimuli [15], [16], [17]. Spinal release of DA has also been shown to activate spinal motor networks involved in locomotion [9], [18], [19], [20]. Altogether, this experimental evidence underscores the role of dopaminergic spinal innervation in the modulation of the sensory and motor processes. It is therefore conceivable that damaged dopaminergic spinal transmission may contribute to RLS symptoms [10], a condition being improved by dopamine replacement therapy [21], [22].

As neurological disorders such as PD are best modeled in non-human primate (NHP) [23], better knowledge of the anatomo-functional organization of the diencephalospinal pathway in the NHP is critical. Therefore, we studied in the normal macaque monkey (i) the regional distribution of DA spinal receptors subtypes using *in situ* hybridization (ISH), (ii) the origin of the dopamine source using a retrograde labeling technique and (iii) the distribution and phenotype of these diencephalic A11 neurons using immunohistochemistry. Finally, the consequences of a 1-methyl-4-phenyl-1, 2, 3, 6-tetrahydropyridine (MPTP)-induced PD syndrome upon the diencephalospinal pathway were studied.

## Materials and Methods

### Animals

Eighteen adult female rhesus monkeys (Macaca mulatta, Cynbiose, Marcy l'Etoile, France) weighing 5 to 9 kg were used for this study. In summary, 13 healthy animals were used: 4 monkeys for ISH and binding procedures, 2 for retrograde tracing study, 4 for stereology and immunohistochemical procedures and 3 for HPLC dosages. In addition, 5 MPTP-treated animals were used: 2 for stereology and immunohistochemical procedures and 3 for HPLC dosages. Experiments were carried out in accordance with European Communities Council Directive of 24 November 1986 (86/609/EEC) for care of laboratory animals in facility accredited by the “Direction des Services Veterinaires” of Rhone area (Department N°69; France). The study design describing the animal use was specifically approved by the VetAgroSup school (Marcy L'Etoile, France) IACUC. Veterinarians skilled in the healthcare and maintenance of non-human primates supervised animal care. All efforts were made to minimise animal suffering. The use of primates was minimised by using an experimental design that permits statistically-significant changes to be demonstrated with the smallest number of animals per group and the smallest number of groups, consistent with scientific rigour. All steps were taken to ameliorate the welfare and to avoid the suffering of the animals in accordance with the “Weatherall report for the use of non-human primates” recommendations. Animals were housed in adjoining individual primate cages (pen area >2 m^2^) allowing social interactions, under controlled conditions of humidity (50%±20% relative humidity), temperature (24°C±2°C) and light (12-hour light/12-hour dark cycles, time lights on 8:00 a.m.). Food and water were available *ad libitum*. Experiments were conducted according to previously published procedures and methods [24], [25].

The animals were euthanized with a sodium pentobarbital overdose (150 mg/kg, i.v.). For the immunohistochemistry procedures, the animals were perfused through the ascending aorta with 2 l of 0.9% saline followed by 3 l of 4% paraformaldehyde in a phosphate buffer (pH 7.4) as fixative. Brains and spinal cords were removed, sliced into frontal sections and placed for 24 h at 4°C in the same fixative. Tissue slices were then rinsed for 48 h at 4°C in 20% sucrose in Tris buffered saline (pH 7.4), frozen in −45°C isopentane and cut into 50 µm frontal sections with a cryostat (Leica). For the ISH and biochemical procedures, brains and spinal cords were freshly frozen in −45°C isopentane and stored at −80°C until use.

### In situ hybridization

The *in situ* hybridization (ISH) procedure was performed as previously described [24], [26] with probes designed to recognize the human D1 DA receptor subtype (2300 pb EcoR1-XbaI fragment; gift from O. Civelli; [27]), the human D2 DA receptor subtype (2300 pb SST1-Hind III fragment; gift from M. Caron; [28]), the human D3 DA receptor subtype (400 pb BamH1 fragment; gift from J.C. Schwartz; [29]), the human D5 DA receptor subtype (1652 pb KpnI-Hind III fragment; gift from M. Caron; [30]) and the human dopamine transporter (DAT) (1200 pb EcoRI fragment; gift from B. Giros; [31]). Radiolabeled antisense and sense 0.25 Kb cRNA probes were prepared by in vitro transcription from the linearized plasmid (0.5 µl, 50 ng) using [^35^S] (Perkin-Elmer; >1,000Ci/mmol) and appropriate RNA polymerase (T7, T3 or SP6; Gibco BRL). Cryostat-cut serial lumbar sections and brain frontal sections (12 µm) were thaw-mounted on gelatin-coated slides. After alkaline hydrolysis to obtain 0.25 kb complementary RNA fragments, the probes were purified on G50-Sephadex and precipitated in sodium acetate (0.1 vol)–absolute ethanol (2.5 vol). Sections were hybridized for 1 night as previously described [24], [26] and then exposed in contact with Biomax film (Kodak) for 10–30 days, dipped into Ilford K5 emulsion, and developed after 8–12 weeks of exposure. The sections were counterstained with hemalun and mounted in Eukitt. Neurons and glial cells were also detected with a toluidine blue counterstain on an adjacent lumbar spinal cord section (L4) in order to provide a baseline appreciation of the neuronal/glial distribution from which the ISH data could be interpreted.

### Dopamine transporter binding

[^125^I] (E)-*N*-(3-iodoprop-2-enyl)-2β-carboxymethyl-3β-(4′-methylphenyl)-nortropane (PE2I) binding (specific activity: 2000 Ci/mmol) was performed as previously described on cryostat-cut serial lumbar and brain sections (12 µm) [32]. The latter were then incubated for 90 min at 25°C with 100 pM [^125^I] PE2I in pH 7.4 phosphate buffer (in mM: NaH_2_PO_4_ 10.14, NaCl 137, KCl 2.7, and KH_2_PO_4_ 1.76). Adjacent sections were incubated in the presence of 100 µM cocaine (Sigma-Aldrich, St. Louis, MO, USA) to define nonspecific binding. After incubation, sections were washed twice for 20 min in phosphate buffer at 4°C and then rinsed for 1 sec in distilled water at 4°C. After drying at room temperature, slides were exposed in contact with Biomax film (Kodak) for 3 days to assess autoradiographically the radioactivity bound to regions of interest.

### Fluoro-Gold Injections

After i.m. premedication with 1 mg/kg diazepam (Valium, Roche), 0.05 mg/kg atropine sulfate (Aguettant) and 10 mg/kg ketamine chlorhydrate (Virbac), animals were intubated and anesthetized with isoflurane (1%). Animals were immobilized in a stereotaxic frame. A laminectomy was performed at cervical vertebra level (C2 to C5) and the dura was carefully opened to expose the spinal cord. The fluorescent retrograde tracer FluoroGold (FG; Sigma-Aldrich) was unilaterally injected (right side; 1 µl/10 min at 4% in 0.9% saline solution; 2 µl/injection) into the dorsal column through a 29 Gauge needle at five locations every 3 mm between C5 and C2 cervical levels at 2–3 mm depth. Then, the dura, the muscles overlying the vertebra and the skin were sutured. Animals were sacrificed 30 days after surgery to allow retrograde FG migration.

### Immunohistochemistry

#### Single labeling

Tyrosine hydroxylase (TH), DAT, dopamine β-hydroxylase (DBH) and aromatic amino acid decarboxylase (AADC) immunoreactivity (IR) was performed within the diencephalon and mesencephalon as previously described in detail [32], [33]. Briefly, free-floating sections were incubated for three nights at room temperature in serum containing TH antibody (1∶10000; Chemicon MAB318, Temecula, CA, USA) or for one night at room temperature in serum containing DAT (1∶1000; Chemicon MAB369), DBH (1∶2000; Chemicon AB1585) or AADC (1∶500; Chemicon AB1569) antibodies diluted in phosphate buffered saline (PBS) with Triton X-100 and 1.5% bovine serum albumin. Sections were then incubated with appropriate biotinylated secondary antibodies (1∶200, GE Healthcare, UK) for 2 hours. Tissue sections were further processed using Vectastain ABC kit (Vector Laboratories, Burlingame, CA, USA) and 3,3-diaminobenzidine tetrahydrochloride (DAB) and nickel for TH, DAT and DBH detection or with the Novared substrate kit for peroxidase (Vector Laboratories) for AADC detection. The sections were mounted on gelatin-coated slides, dried, counterstained with neutral red, dehydrated in graduated concentrations of ethanol, cleared in xylene and mounted in Eukitt.

#### Double labeling

Double fluorescent labeling was performed to determine the distribution of TH- and AADC-positive neurons, TH- and Calbindin-D28k-(CALB, using anti-CALB antibody, 1∶1000, Sigma Aldrich) positive neurons and TH- and FG-positive neurons. FG labeling was enhanced using a FG antibody (1: 5000; Chemicon MAB153) [34]. Following 1 hour of incubation with blocking solution (PBS with 3% normal goat serum, 0.3% bovine albumin serum and 0.05% saponin), the sections were incubated overnight at room temperature with appropriate combinations of two primary antibodies raised in different donor species. Immunoreactions were then visualized by appropriate secondary species-specific antibodies labeled with Alexa Fluor 488 and Alexa Fluor 568 (1∶400, Invitrogen, Carlsbad, CA, USA) after 2 hours of incubation. Sections were incubated for 5 minutes with the autofluorescence eliminator kit (Chemicon) and then coverslipped with Vectashield mounting medium with DAPI (Vector Laboratories).

For unbiased stereological cell counting of TH-, FG- and TH-FG-immunopositive neurons, we opted for a combination of two different colored chromogens for peroxidase. Briefly, free-floating sections were first incubated with FG antibody for one night at room temperature and then processed for IR revelation as described above (using appropriate secondary antibody, GE Healthcare, UK), except for the substrate kit for peroxidase. Indeed, we used an SG substrate kit for peroxidase (Vector Laboratories) that stained the FG-positive neurons blue-gray. After FG neuron revelation, sections were first incubated with an avidin/biotin blocking kit (Vector Laboratories) and then re-incubated with TH antibody (1∶10000) for one night at room temperature. TH-IR was revealed with the NovaRed substrate kit for peroxidase (Vector Laboratories) that stained the TH-positive neurons red. Thus, double-stained neurons positive for TH and FG were labeled in a blue-red combination. The sections were mounted on gelatin-coated slides, dried, dehydrated in graduated concentrations of ethanol, cleared in xylene and mounted in Eukitt.

### Parkinsonism induction

MPTP is a well known dopaminergic specific neurotoxin used to model PD in animals, notably in the NHP. As nothing is know about a potential involvement of the A11 pathway in PD, we sought to investigate the putative toxic effect of the neurotoxin MPTP on A11 neurons. To this end, a total of 5 monkeys received 0.5 mg/kg (i.v.) of MPTP (Sigma-Aldrich) until the development of parkinsonism following a sub-acute intoxication paradigm as previously described [35].

### Determination of spinal dopamine and its metabolite concentrations

Dopamine and its metabolite concentrations after MPTP intoxication were measured in lumbar spinal cord and dorsolateral striatum of 3 MPTP-treated animals and 3 controls. The levels of dopamine, 3,4-dihydroxyphenylacetic acid (DOPAC) and homovanillic acid (HVA) were measured by a standard HPLC technique with electrochemical detection, the detection limit of DA being 0.1 fmol/µl. The samples were injected with an autosampler (SIL-10AD Shimadzu, Japan) and separated by HPLC on a reverse phase column (Luna 5 µ C18(2) 150×4.6 mm, Phenomenex, UK) with a flow rate of 1 ml/min. The mobile phase consisted of 75 mM sodium dihydrogen phosphate, 0.274 mM ethylenediaminetetraacetic acid (disodium salt), 1.4 mM 1-octanesulfonic acid (sodium salt), 10% acetonitrile and the pH was adjusted to 3 with phosphoric acid. Dopamine and its metabolites were quantified electrochemically by a dual-carbon electrode high sensitivity analytical cell (Model 5011, ESA, USA). The potential of the two electrodes was 100 mV and–350 mV. Sensitivity was set at 100 nA/V with an electrochemical detector (Coulochem II, ESA, USA). The chromatograms were recorded with a chromatographic data system (Class vp5.0, Shimadzu, Japan) and quantified by determination of peak areas in relation to standard.

### Stereological cell counts

Unbiased stereological cell counting of A9 (substantia nigra; SN), A10 (ventral tegmental area; VTA) and A11 groups was performed in normal (n = 4) and MPTP-intoxicated (n = 2) animals as previously described [36]. Every fourth section of hypothalamus and mesencephalon was processed for TH-IR. Stereological sampling was performed using a computer-assisted image analysis system (Mercator, ExploraNova, La Rochelle, France) coupled to a Leica DM-6000B microscope. A11 was delineated rostro-caudally at 5x objective using anatomical landmarks (in the posterior hypothalamus, dorsal to the mammillary bodies, immediately lateral to the third ventricle and medial to the mammilotegmental tract). A9 and A10 were delineated as previously described [32], [36]. A random sampling of 100 µm counting frame size and 100 µm grid size was applied. Counting of A9, A10 and A11 neurons was performed at 40x objective to ensure anatomical accuracy in the whole A9, A10 and A11 areas. Guard zones of 1.5 µm ensured the exclusion of lost profiles on the top and bottom of the section sampled. The number of neurons in the A9, A10 and A11 groups was estimated using the optical fractionator method [37], which is unaffected by changes in the volume of reference of the structure sampled and is thus suitable for estimating the number of neurons in brain nuclei that lack well defined anatomical boundaries. The total number of TH-IR neurons in the A9, A10 and A11 areas was calculated based on the following formula: N = Q x (1/ssf) x (1/asf) x (t/h), where N is the estimate of the total number of cells, Q is the number of objects counted, ssf is the section sampling fraction, asf is the area sampling fraction, and t/h is the actual section thickness divided by the height of the dissector. Between 80 and 300 objects were counted to generate the stereological estimates. All cell counts were performed by an investigator blind to the animal experimental status. Following the same stereological counting protocol, neurons stained for TH and double-stained neurons for TH and FG were quantified within the A11 area of the monkeys injected with the retrograde tracer FG. Three categories of neurons were counted: TH-IR neurons labeled in red, FG-IR neurons labeled in blue and double-stained neurons for FG- and TH-labeled in a blue-red combination.

### Statistical analysis

Unpaired *t*-tests followed by Welch's correction using GraphPad Prism 4 software were applied to compare mean DA and its metabolites concentrations in controls and in MPTP animals and to compare stereological counts in controls and in MPTP animals. Data are shown as mean ± standard deviation. Statistical significance was considered at a probability (*P*) value ≤0.05.

## Results

### Topographic distribution of dopaminergic receptors within the lumbar spinal cord

**Figure 1:**
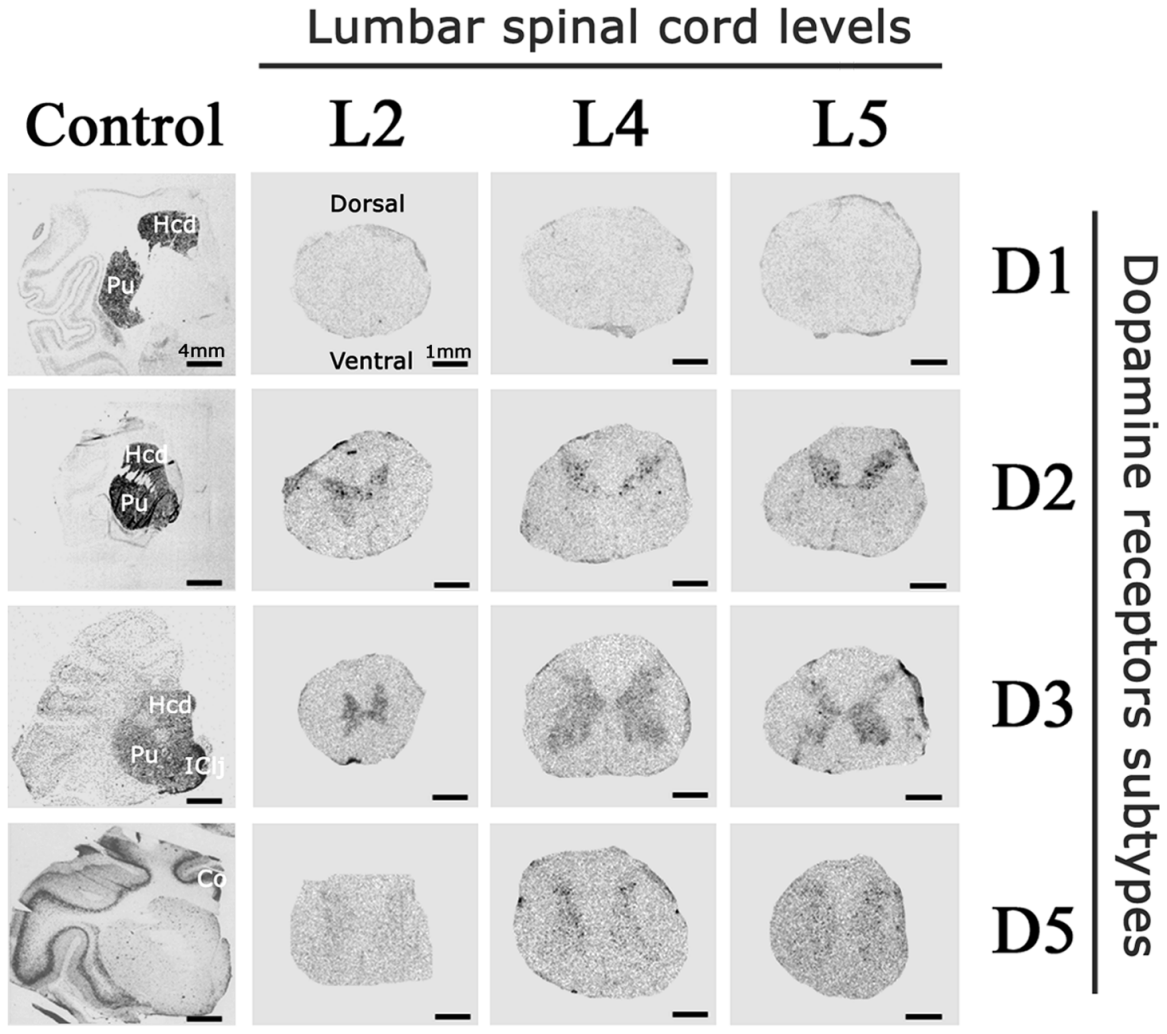
Macroscopic detection of dopamine receptors within the lumbar cord of non-human primate. Representative film autoradiograms after radioactive *in situ* hybridization targeted against the mRNA DA receptors and transporter in lumbar spinal cord transverse sections at different levels (L2, L4 and L5) and in frontal brain sections (positive control). Note that D2 and D3 subtypes are the most expressed DA receptors: D2 receptors were more highly expressed in dorsal horn of the spinal cord whereas D3 receptors showed lower levels of expression with much wider distribution within the gray matter of the spinal cord. Expression of the D1 subtype was not detected whereas the D5 subtype was poorly expressed. Abbreviations: Hcd = Head of Caudate nucleus; Pu = Putamen; IClj = Islands of Calleja; Co = Cortex.

Radiolabeled antisense cRNA probes were used to determine the topographic distribution of the DA receptor subtypes within the lumbar cord. Figure 1 provides representative transverse sections of lumbar spinal cord labeled with antisense probes for the various DA receptors. To ensure the efficacy and specificity of antisense radiolabeled cRNA probes, ISH was also performed in forebrain frontal sections. As expected, D1 and D2 receptor subtypes were mainly expressed within the striatum and cortex, D3 mainly expressed within the ventral striatum and the Islands of Calleja and D5 mainly expressed within the cortex (Fig. 1) [38]. These results confirm the specificity of the cRNA probes for each DA receptor subtype.

In the primate lumbar spinal cord, the labeling appeared restricted to the interior gray matter and remained unchanged whatever the lumbar spinal level (L2, L4 or L5). Overall, D2, D3 and D5 receptor subtypes were expressed in the lumbar spinal cord sections. By contrast, the D1 receptor autoradiograms did not show any positive signal in the NHP spinal cord (Fig. 1).

**Figure 2:**
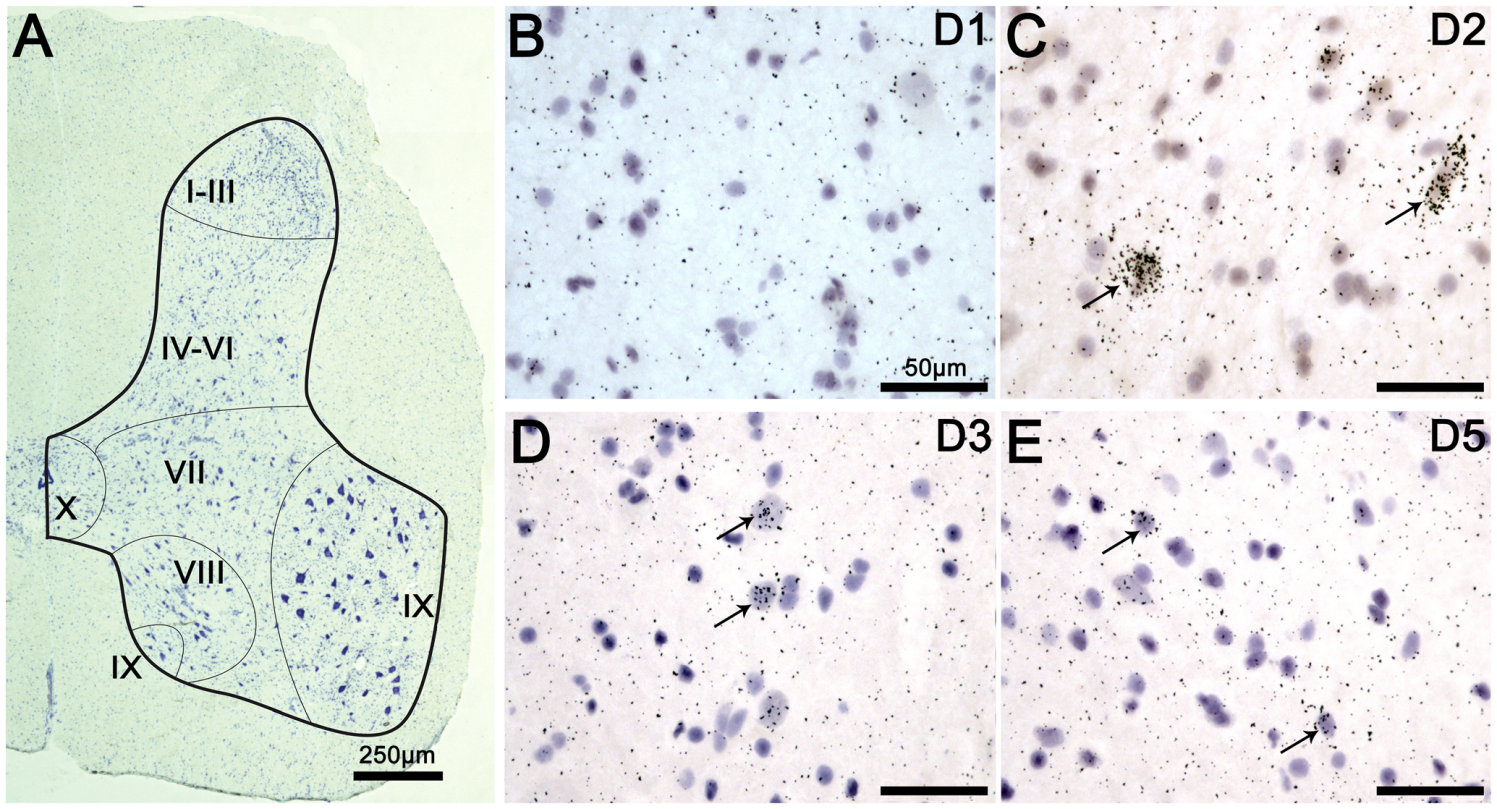
Microscopic detection of D2, D3 and D5 dopaminergic receptors in the lumbar cord. **A.** Reconstructed micrograph showing a representative distribution of neurons and glial cells in a section from the lumbar spinal cord (L4). The distribution of neurons and glial cells is shown with toluidine blue staining. Surrounding white and internal gray matter are delineated with a thick black line. Division of spinal cord into laminae is approximate and separated with fine black lines according to the Rexed laminae description. Note that in laminae I-III, neuronal cell body diameters are generally the smallest. In laminae IX, neuronal cells bodies are generally the biggest (probably corresponding to motoneurons). B. Illustrative brightfield micrograph showing the detection of D1 labeling in the dorsal part of a lumbar spinal cord section. Note the lack of D1 positive cells. **C.** Illustrative brightfield micrograph showing the detection of D2 labeling in the dorsal part of a lumbar spinal cord section. The arrows point to typical positive cells for D2 labeling. **D.** Illustrative brightfield micrograph showing the detection of D3 labeling in the ventral part of a lumbar spinal cord section. The arrows point to typical positive cells for D3 labeling. **E.** Illustrative brightfield micrograph showing the detection of D5 labeling in the dorsal part of lumbar spinal cord section. The arrows point to typical positive cells for D5 labeling. Note that D2 labeling was mainly found in laminae I to VI, that the D3 labeling showed a wider distribution in laminae I to X and that the D5 labeling was mainly found in laminae I to III.

The macroscopical,analysis showed that the D2 receptor distribution is mainly located within the dorsal horns of the spinal cord. The DA D3 receptor signal showed a much wider distribution within the gray matter of the spinal cord. The D5 receptors were predominantly distributed within the dorsal horns. The D5 subtype signal appeared to be weaker than the D2 and D3 signals. Prior to the microscopic analysis of the distribution of DA receptors, we first undertook detection of neurons and glial cells in adjacent sections to provide a baseline appreciation of the neuronal/glial distribution from which the ISH data could be interpreted. In the spinal section provided in Figure 2A, the gray matter has been outlined together with its classical subdivisions in Rexed laminae (I-III; IV-VI, VII, VIII, IX, and X) [39]. The microscopic analysis of spinal cord sections confirmed the macroscopic regional distribution of DA receptors within the cord. At the cellular level, the analysis confirmed the lack of D1 expression, as illustrated in Figure 2B. The detection of D2 labeling in spinal cord sections showed that D2 receptors were intensely expressed in the dorsal horns mainly within the laminae I to VI, as illustrated in Figure 2C. The detection of D3 labeling showed that the cells expressing D3 receptors were more homogeneously distributed within the gray matter, i.e. laminae I to X, as illustrated in Figure 2D. Lastly, detection of D5 labeling confirmed that the cells expressing this receptor were mainly located in the dorsal laminae I to III of the lumbar spinal cord, as also illustrated in Figure 2E. Several D5 positives cells were also found in the spinal ventral horns.

### TH immunohistochemistry within the hypothalamus: comparison with TH-IR distribution in human

**Figure 3:**
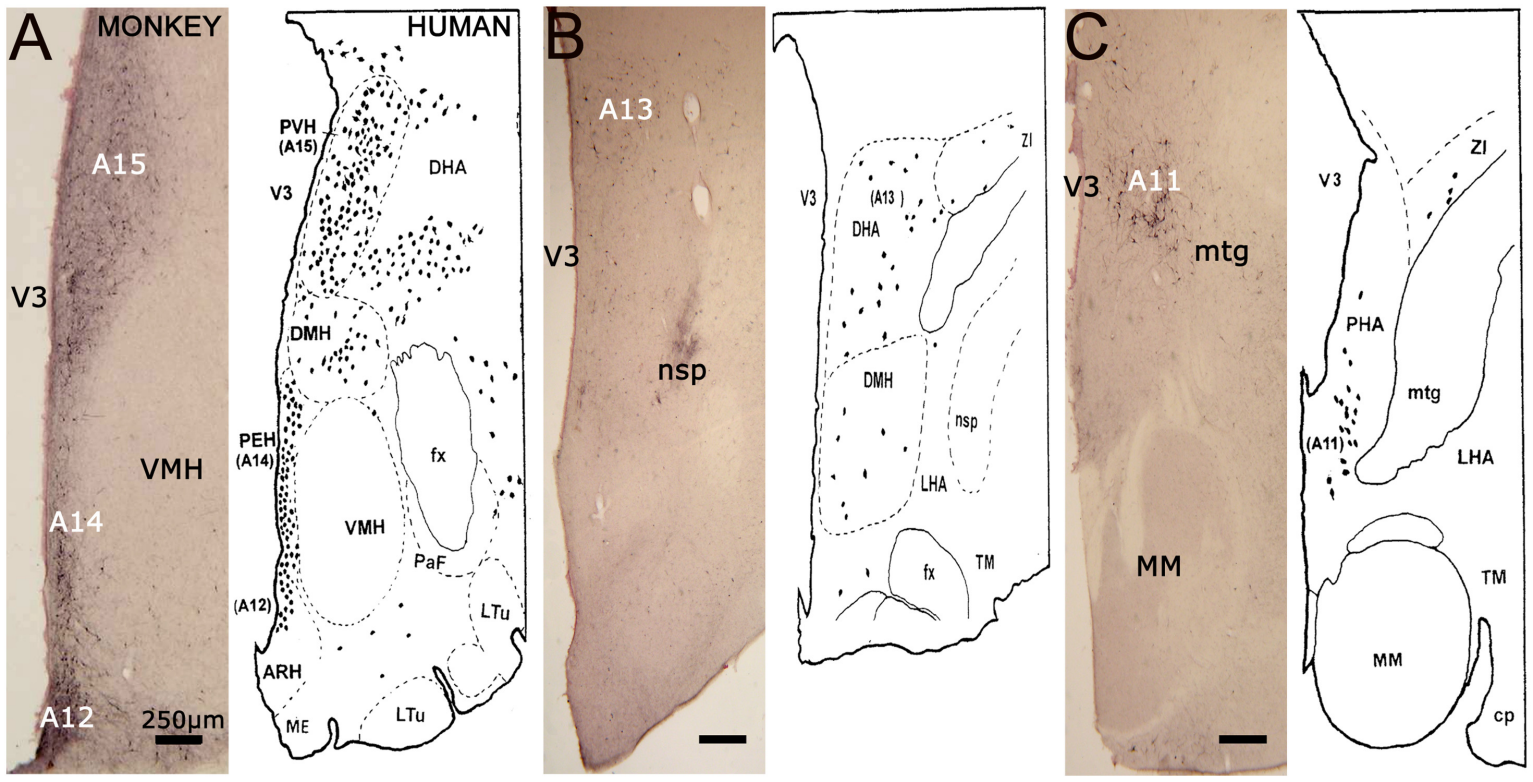
Characterization of tyrosine hydroxylase-positive neurons within the diencephalon in the non-human primate compared to human. **A.** At the anterior hypothalamus level (AC-3 mm) where TH-IR regions A12, A14 and A15 are delineated. **B.** At the medial hypothalamus (AC-4 mm) where region A13 is delineated. **C.** At the posterior hypothalamus level (AC-5 mm) where the TH-IR A11 region is delineated. Note the remarkable concordance between NHP and human TH-IR distributions. Representative drawings of the human diencephalon are taken from Kitahama et al., 1998 with the permission of Elsevier (License N°2361961459060). Abbreviations: AC = Anterior Commissure; ARH = Arcuate Hypothalamic Nucleus; cp = Cerebral Peduncle; DHA = Dorsal Hypothalamic Area; DMH = Dorsomedial Hypothalamic Nucleus; fx = fornix; LHA = Lateral hypothalamic Area; Ltu = Lateral tuberal nucleus; nsp = nigrostriatal dopaminergic pathway; PaF = Parafornical nucleus; PEH = Periventricular Hypothalamic nucleus; PHA = Posterior Hypothalamic Area; TM = Tuberomammillary nucleus; V3 = Third Ventricle; VMH = Ventral Medial Hypothalamus; ZI = Zona Incerta.

Figure 3 provides a representative example of TH-IR within the anterior (Fig. 3A), medial (Fig. 3B) and posterior (Fig. 3C) aspect of the NHP hypothalamus compared with the single available description of hypothalamic TH-IR distribution in human [40].

TH-IR distribution in the anterior hypothalamus matches with that described in human. TH-IR cells were indeed seen in the dorsal and medial parts of the hypothalamus at the level of the caudal portion of the ventromedial hypothalamic nucleus (VMH). In the arcuate nucleus (ARH), TH-IR neurons (the human A12 group) were packed ventrally in continuity with a periventricular group. This periventricular group, which was composed of numerous small (15–20 µm) TH-IR cell bodies vertically arranged along a narrow strip extending across the lower half of the third ventricle, probably corresponds to the human A14 group. Finally, the paraventricular hypothalamic nucleus (PVH), which comprised numerous large aggregated TH-IR neurons, likely corresponds to the human A15 group (Fig. 3A).

Between the anterior and posterior hypothalamus, medium-sized TH-IR cell bodies (25–35 µm) were distributed in the dorsal hypothalamic area and probably correspond to the human A13 group (Fig. 3B).

In the posterior hypothalamus, TH-IR cell bodies were present in the dorsal portion between the mammilotegmental fasciculus and the third ventricle. They were more abundant at the level of the mammilary nuclei. These medium-sized neurons were oval or fusiform in shape and gave rise to two or three main processes that were vertically oriented. These neurons are believed to be the A11 group described in human (Fig. 3C). Stereological counts showed that the A11 neuron group is composed by 4002±501 TH-IR neurons per side.

### Localization of diencephalospinal pathway origin by retrograde tracing with FG

**Figure 4:**
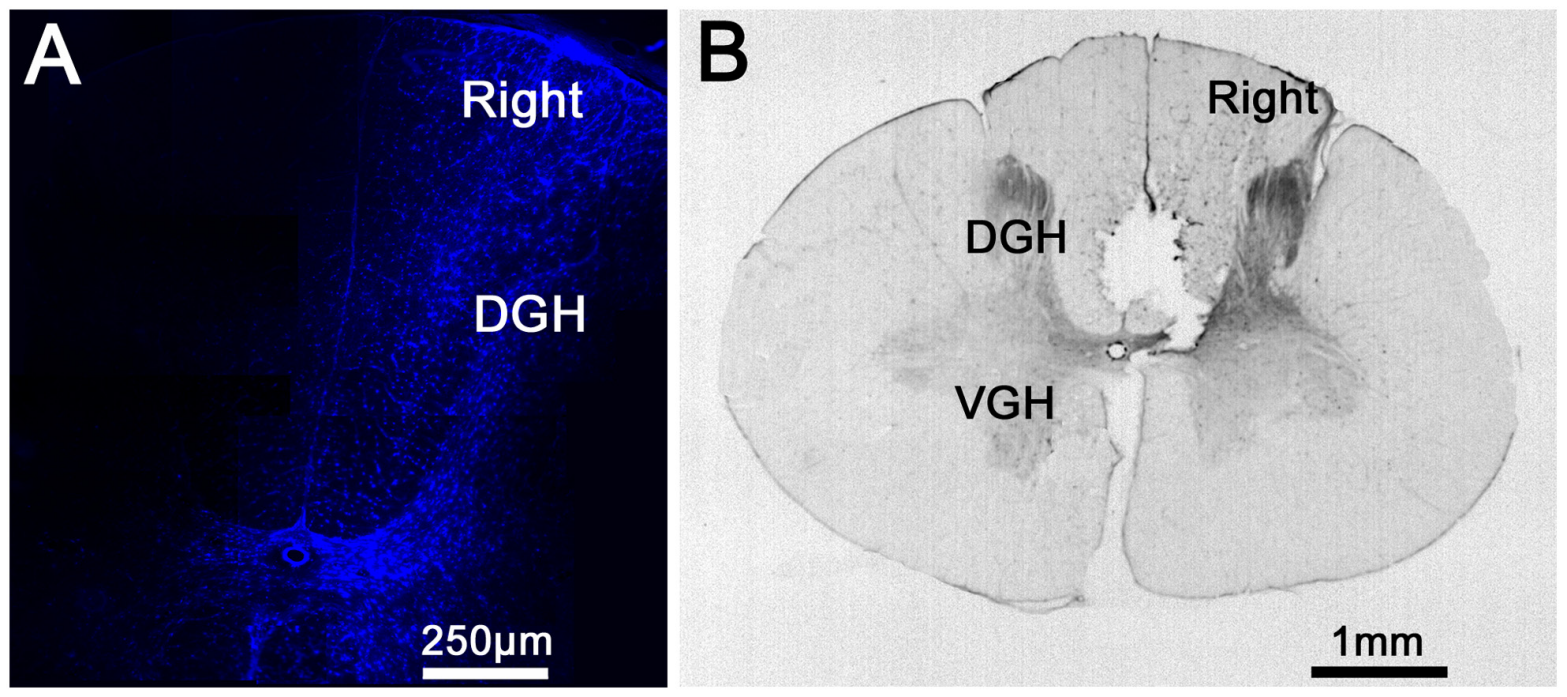
Histological analyses of FluoroGold injections into the lumbar spinal cord. **A.** Visualization of FG labeling by fluorescence microscope providing ultraviolet excitation light. **B.** Visualization of FG labeling by immunohistochemistry directed against FG. Note that FG was injected into the right dorsal horn of the cervical spinal cord and that there was a slight diffusion of the marker within the contralateral side. Abbreviations: DGH = Dorsal Gray Horn; VGH = Ventral Gray Horn.

Histological analyses of FG injection sites within the cervical cord by fluorescence microscopy, providing ultraviolet excitation light to directly visualize FG (Fig. 4A) or by immunohistochemistry directed towards FG (Fig. 4B), confirmed that FG was injected unilaterally into the cervical dorsal column of the spinal cord. Nevertheless, a spinal diffusion of FG towards the contralateral side and/or the central aspect of the cord was observed.

**Figure 5:**
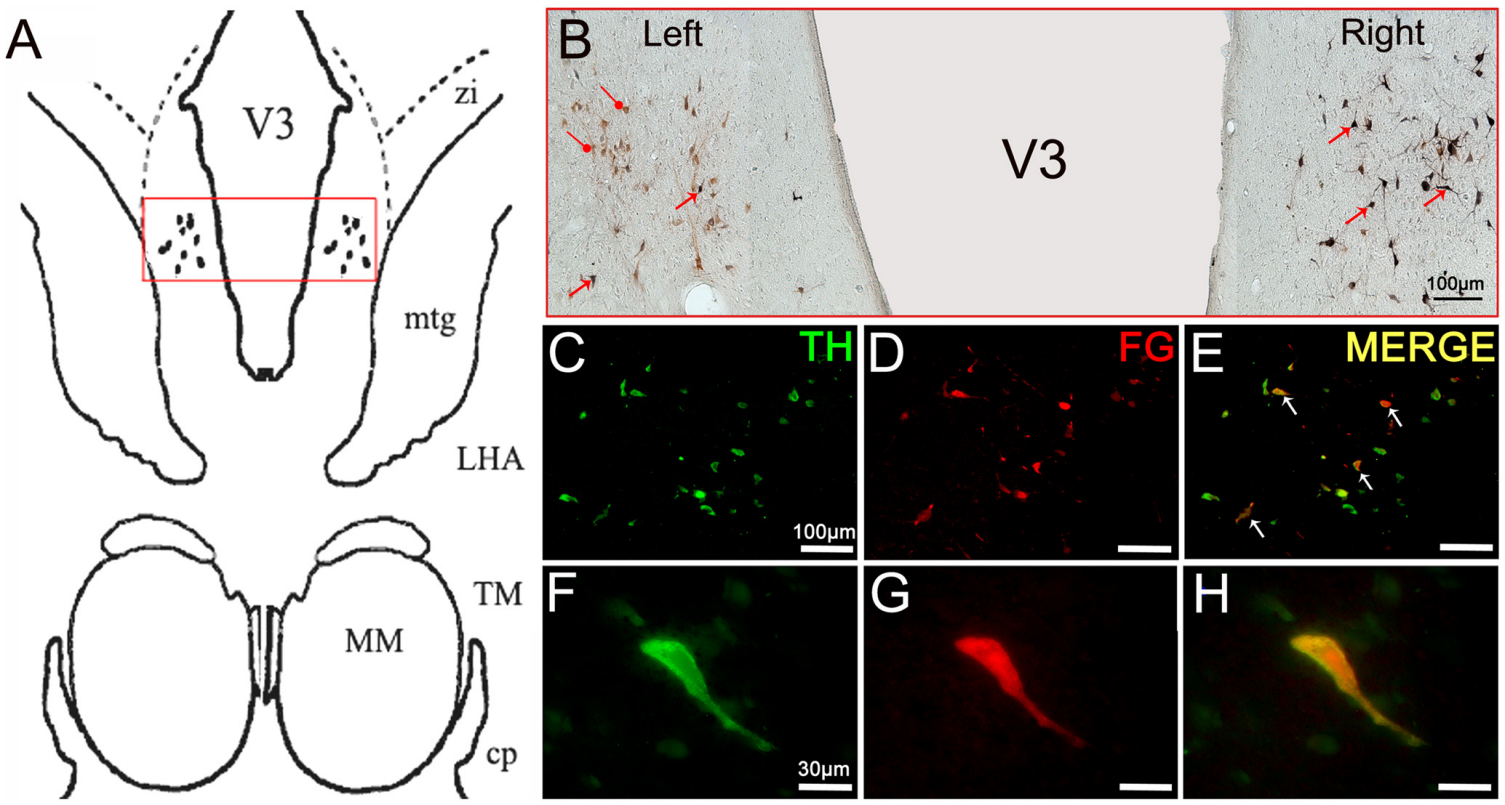
Retrograde labeling of A11 neurons projecting to the spinal cord. **A.** Schematic representation of the hypothalamic A11 area in which TH-IR neurons were reached by FluoroGold. The dots represent the localization of TH-IR A11 neurons. The red frame represents the area where representative micrographs were taken. **B.** Representative micrograph showing a reconstructed overview of the TH immunopositive and FG retrograde labeled cells in the A11 group. Immunoreactivity was revealed with Novared kit for TH neurons (Red) and with SG kit for FG neurons (Blue). Double-stained neurons (TH-FG) were labeled in a blue-red combination (i.e. black). The black arrows point to typical double-stained cells. The round heads arrows point to TH-stained neurons. Note that no single FG-stained neurons were found and that the majority of double-stained neurons are located on the ipsilateral side of spinal injections (right) **C–H**: Representative double-fluorescent immunostaining of TH (green) and FG (red) obtained under a confocal laser-scanning microscope in the A11 posterior hypothalamic group. The white arrows point to typical double-stained cells. Note the colocalization between the TH-positive and FG-positive neurons within the A11 region. Abbreviations: cp = Cerebral Peduncle; FG = FluoroGold; MM = Medial Mammillary nucleus; LHA = Lateral Hypothalamic Area; TH = Tyrosine Hydroxylase; TM = Tuberomammillary nucleus; V3 = Third Ventricle; ZI = Zona Incerta.

In the diencephalon, the retrogradely-labeled neurons showed typical cytoplasmic granular staining. As the main focus of our work was the A11 area, we did not thoroughly explore the noradrenergic and serotoninergic supra-spinal descending systems. However, several FG-labeled cells were seen in the locus coeruleus and in the dorsal raphe, thus confirming the diffuse uptake and retrograde transport of FG. In the hypothalamus, FG-labeled cells were located mostly in the posterior hypothalamus between the third ventricle and the mammilotegmental fasciculus (Fig. 5A).

Double staining of TH and FG showed that the double-stained neurons were located within the A11 group (Fig. 5B–H), while very rare double-stained neurons were also found within the A13 group. The TH-labeled neurons did not differ morphologically from the TH-FG-labeled neurons. The double-stained neurons were located throughout the entire rostro-caudal extension of the A11 area and were mainly located on the side of the FG injection, although a sparse contralateral labeling was also observed. Stereological counting of the FG-TH-labeled neurons identified 3120 neurons in the ipsilateral A11 group (72.2% of total TH-IR neurons) and only 296 neurons (8.0% of total TH-IR neurons) in the contralateral A11 group (Fig. 5B). Therefore, A11 neurons mainly project ipsilaterally within the cord.

### Phenotypic characterization of posterior hypothalamic TH-IR neurons

**Figure 6:**
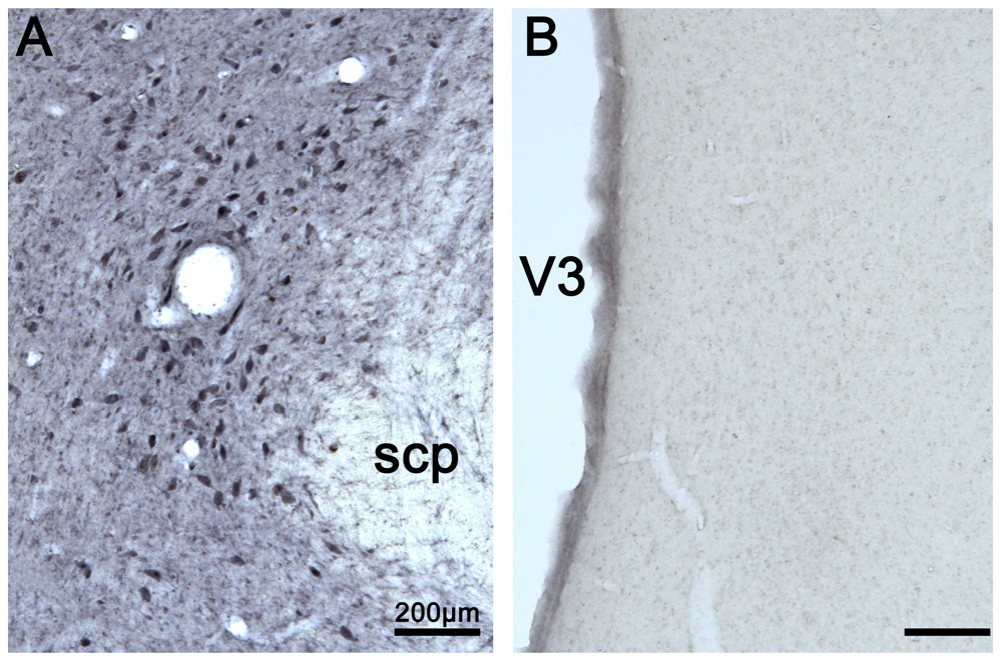
Dopamine beta-hydroxylase (DBH) expression in the locus coeruleus (A) and the posterior hypothalamus (B). Note the lack of DBH labeling in the posterior hypothalamus (A11 area). Abbreviations: scp = superior cerebellar peduncle; V3 = Third Ventricle.

To further characterize the phenotype of the A11 TH-IR neurons in the NHP, we first performed DBH-IR in the posterior hypothalamus and in the locus coeruleus (positive control). As expected, DBH-positive noradrenergic neurons were found in the locus coeruleus (Fig. 6A) [41]. In the posterior hypothalamus, no DBH-positive neurons were noted, thus indicating the absence of noradrenergic neurons within this region (Fig. 6B).

**Figure 7:**
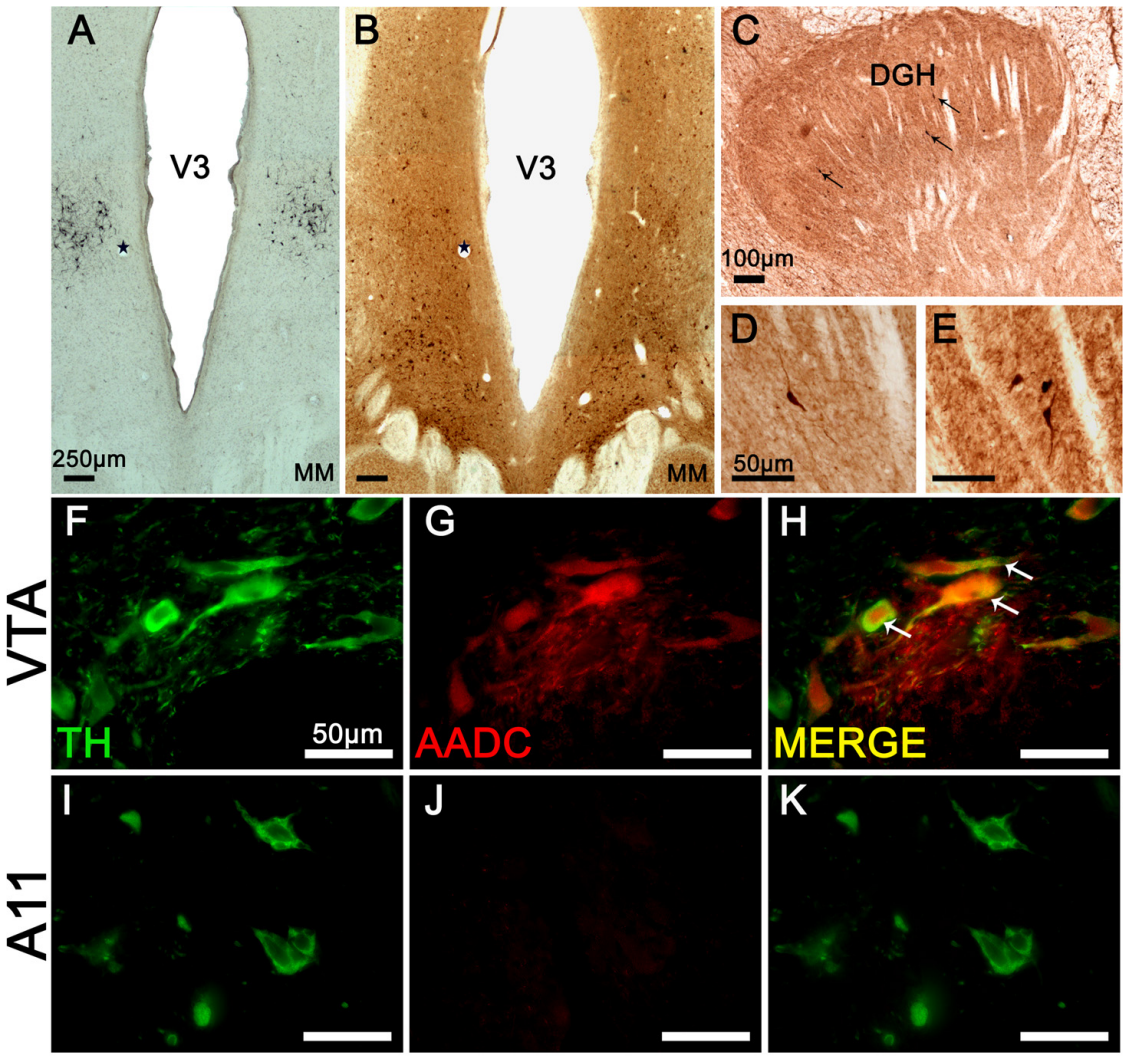
Aromatic aminoacid decarboxylase (AADC) expression in posterior hypothalamus and spinal cord. **A–B**: Immunohistochemistry targeted against TH (**A**) and AADC (**B**) processed on adjacent sections of the posterior hypothalamus. Note the lack of AADC labeling in the region of TH-IR A11 neurons. Stars label the same blood vessel profiles in adjacent sections (A and B). **C–E**: Immunohistochemistry targeted against AADC within the dorsal horn aspect of the spinal cord. The black arrows point to typical AADC positive neurons. **F–K**: Double-fluorescent immunostaining of TH (green) and AADC (red) obtained under a confocal laser-scanning microscope in a section through the VTA (**F–H**) and A11 (**I–K**) groups. The white arrows point to typical double-stained cells in VTA. Note the absence of AADC-positive neurons within the A11 region. Abbreviations: AADC = Aromatic Aminoacid Decarboxylase; DGH = Dorsal Gray Horn; MM = Mammillary nucleus; TH = Tyrosine Hydroxylase; V3 = Third Ventricle.

In addition, AADC-IR was performed within the posterior hypothalamus. Interestingly, AADC-IR positive neurons (Fig. 7B) did not display a similar distribution to that of the TH-IR cells in the posterior hypothalamus (A11 area) (Fig. 7A). Indeed, AADC-positive neurons were mainly found in a more ventral position than the A11 TH-IR neurons, in a region corresponding to the supramammilary nucleus. Very few AADC-positive neurons were located in A11 group area, suggesting that the TH-IR A11 neurons were exclusively monoenzymatic. This was confirmed by the fluorescent double labeling against TH and AADC showing that AADC-positive neurons were not colocalized with TH-positive neurons within the A11 group (Fig. 7F–K). In spinal cord, we also found a few AADC-positive neurons strictly located in the dorsal horn, within Rexed laminae I to VI (Fig. 7C–E).

**Figure 8:**
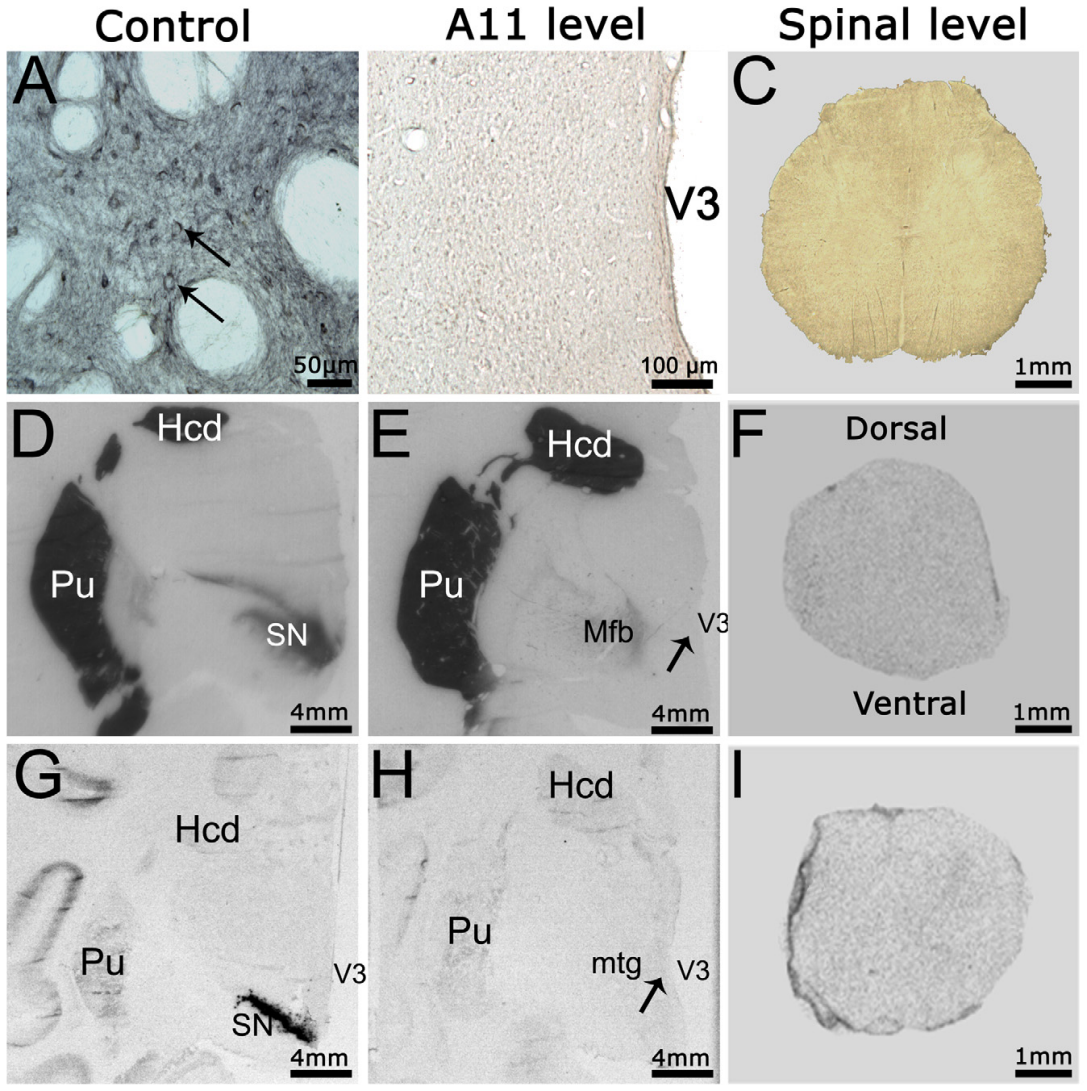
Absence of dopamine transporter expression in the diencephalospinal pathway. **A–C**: Immunohistochemistry targeted against the DAT. **A.** Representative micrograph of DAT labeling positive neurons in the ventral tegmental area. The arrows point to typical immunopositive neurons. **B.** Representative micrograph showing the absence of DAT labeling at the posterior hypothalamus level. **C.** Representative micrograph showing the absence of DAT labeling in a lumbar spinal section. **D–F**: Representative DAT binding autoradiographs in the substantia nigra (**D**), posterior hypothalamus (**E**) and lumbar spinal cord (**F**). Note the absence of DAT expression in the posterior hypothalamus and spinal cord, in contrast with the intense expression in the substantia nigra and striatum (positive controls). The arrow points to the approximate location of A11 area. **G–I**: Film autoradiograms after radioactive i*n situ* hybridization targeting the DAT mRNAs in the substantia nigra (**G**), posterior hypothalamus (**H**) and lumbar spinal cord (**I**). Note the absence of DAT mRNA expression in the posterior hypothalamus and spinal cord, in contrast with the intense expression in the substantia nigra (positive control). The arrow points to the approximate location of A11 area. Abbreviations: Hcd = Head of Caudate nucleus; Mfb = Medial forebrain bundle; MM = Medial Mammillary nucleus; mtg = Mammilotegmental fasciculus; Pu = Putamen; SN = Substantia Nigra; V3 = Third Ventricle.

Finally, we used a combination of DAT immunohistochemistry (Fig. 8A–C), DAT binding (Fig. 8D–F) and DAT ISH (Fig. 8G–I) on brain and spinal cord sections to determine whether TH-IR A11 neurons express DAT. The three techniques showed that neurons in the posterior hypothalamus do not express DAT. This absence of DAT expression was confirmed in the lumbar spinal cord where positive fibers (or neurons) were not detected by either technique (Fig. 8C, F, I). The absence of DAT expression was not due to a lack of specificity of the techniques used since SN neurons serving as positive control were clearly positive for DAT-IR (Fig. 8A), DAT binding (Fig. 8D) and DAT mRNA expression (Fig. 8G).

### Effects of the neurotoxin MPTP on A11 neurons and DA concentration in spinal cord

As it has been shown that limb rigidity in patients with PD points to sensorimotor processing damage resulting from a change in their descending monoaminergic inhibitory control [42], we sought to determine the effect of a pro-parkinsonian neurotoxin on the diencephalospinal pathway despite the absence of DAT expression.

**Figure 9:**
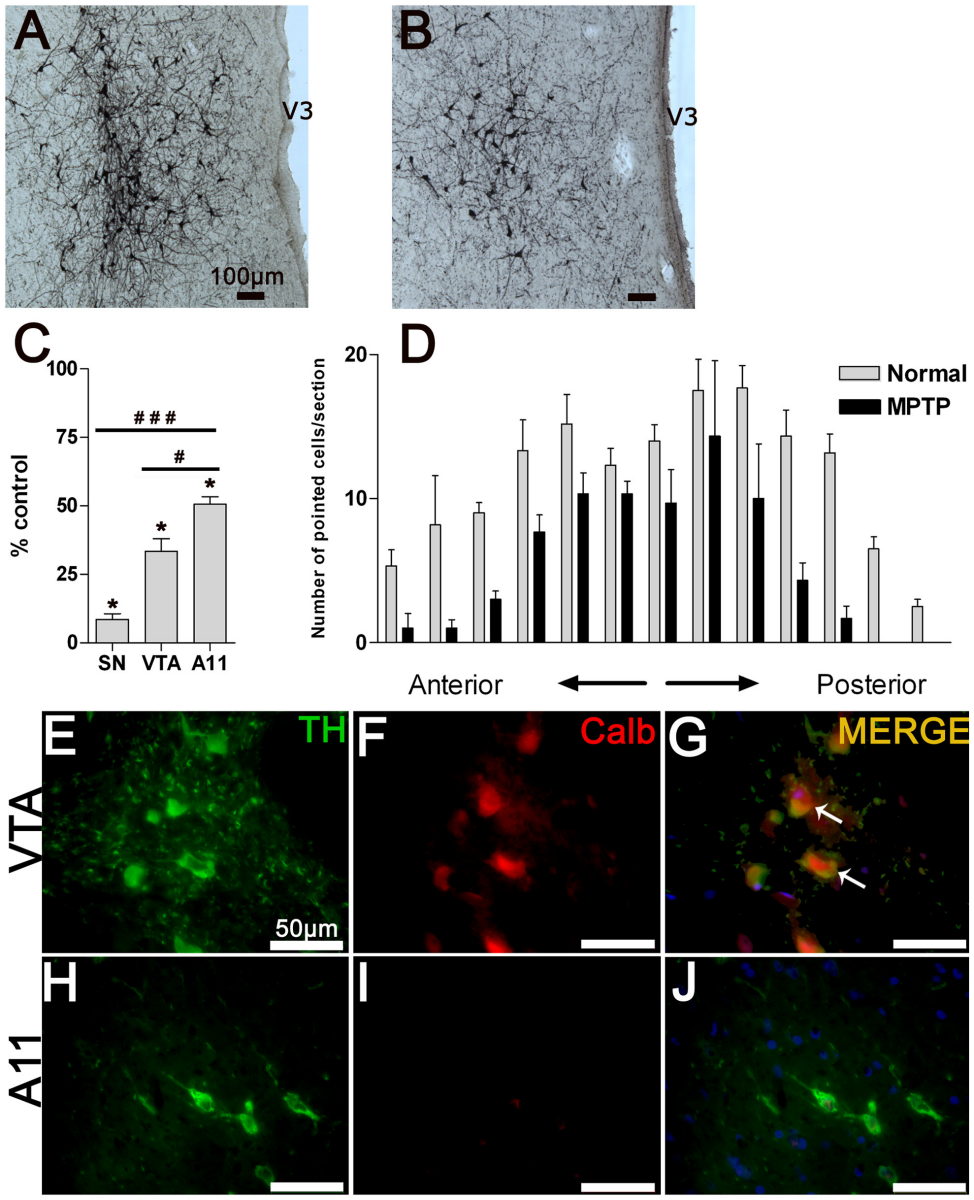
Effect of the neurotoxin MPTP on hypothalamic A11 neurons. **A.** Representative microphotograph of TH-IR A11 neurons in a control animal. **B.** Representative microphotograph of TH-IR A11 neurons in an MPTP-intoxicated animal with full parkinsonism. Note the large cell loss within the A11 area. **C.** Mean (±SD) percentage of total number of TH-IR neurons remaining following MPTP intoxication within the substantia nigra, ventral tegmental area and A11 group (*P<0.0005, comparison between MPTP-treated animals (n = 2) and control animals (n = 4); ^#^ *P*<0.02 and ^###^ *P*<0.0003, comparison between dopaminergic groups following MPTP intoxication, two-tailed *P* value, unpaired *t*-test). Note the difference in cell loss following MPTP intoxication between the A11 group and the various DA regions. **D.** TH-IR counted cells were mapped in individual sections from anterior to posterior hypothalamic A11 area with 200 µm section intervals. Note the general cell loss at different levels of the A11 area in MPTP-treated animals (n = 2) compared to controls (n = 4). **E–J**: Representative micrograph of TH (green) and CALB (red) double fluorescent immunostaining obtained under a confocal laser-scanning microscope in VTA (**E–G**) and A11 (**H–J**) sections. The white arrows point to typical double-stained neurons. Note the absence of CALB-positive neurons within the A11 region. Abbreviations: Calb = Calbindin 28 k; TH = Tyrosine Hydroxylase; V3 = Third Ventricle.

As expected from previous works [32], [36], a significant loss of TH-IR neurons was observed in the A9 (SN) and A10 (VTA) dopaminergic groups following the systemic administration of MPTP until the development of full parkinsonian symptom (respectively, 91.4±3.62% of neuronal loss compared to control, *P*<0.001 and 66.5±4.5% of neuronal loss compared to control, *P*<0.001; unpaired *t*-test). Moreover, a significant cell loss within the A11 group was found following MPTP intoxication (mean number of neurons in control animals compared with MPTP-treated animals, 4002±501 vs. 2027±110, *P*<0.001; unpaired *t*-test) (Fig. 9A–C). Neuronal cell counts throughout the hypothalamic antero-posterior axis showed that this neuronal loss was uniform (Fig. 9D). We noted significant sensitivity differences between the A9, A10 and A11 regions following MPTP intoxication (neuronal loss in A9 compared with A11 group, 91.4±3.6% vs. 49.39±4.8%, P<0.001 and neuronal loss in A10 compared with A11 group, 66.5±4.5% vs. 49.39±4.8%, *P* = 0.011; unpaired *t*-test). As calcium binding protein calbindin-D28k (CALB) neuronal expression reduces the vulnerability to MPTP-induced neurodegeneration [43], [44], we performed a double labeling against TH and CALB within the A11 and VTA (positive control) regions. As expected, positive TH-CALB neurons were found in VTA (Fig. 8E–G) [45]. However, the TH-IR neurons of the A11 group did not express CALB (Fig. 8H–J).

**Figure 10:**
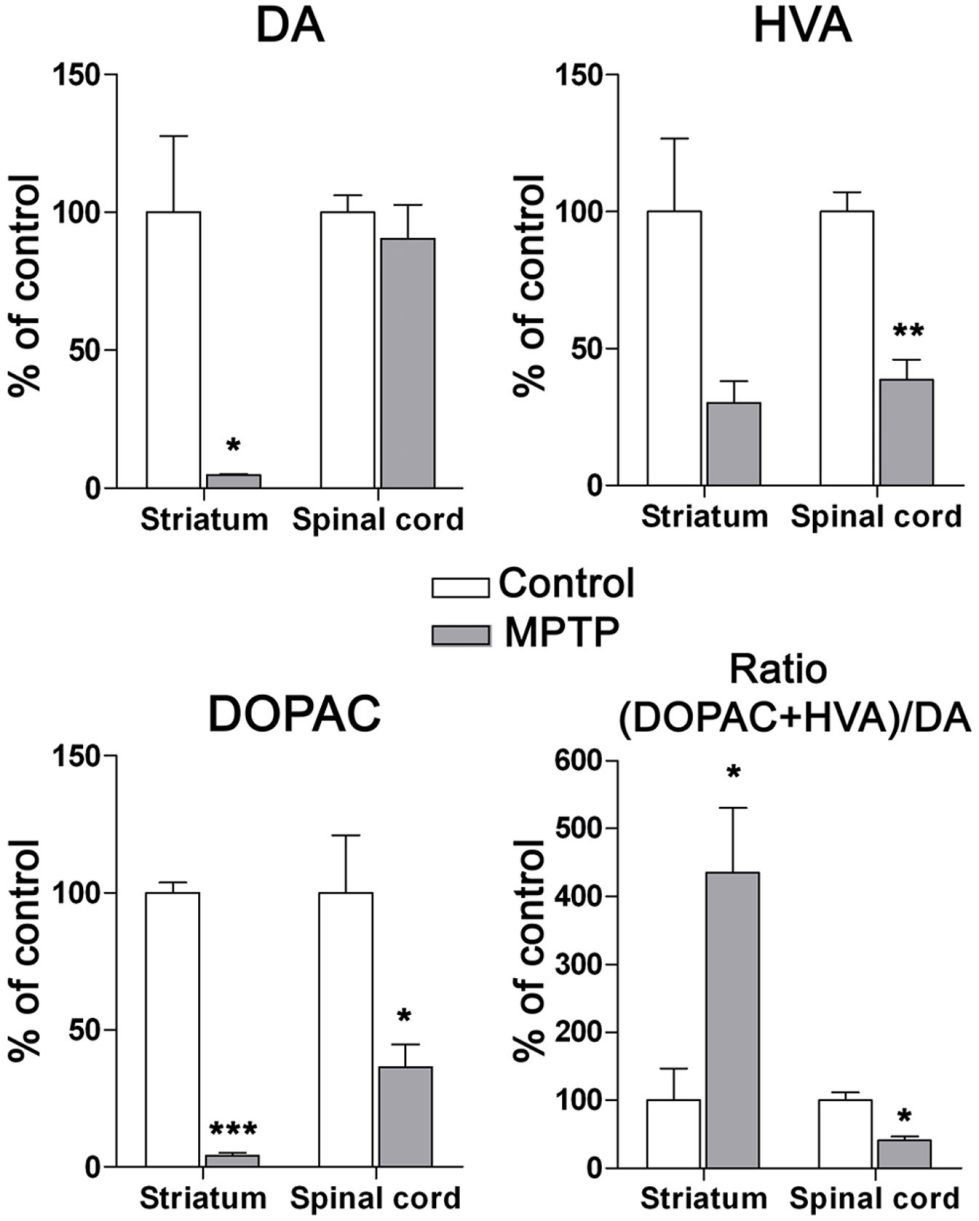
HPLC detection of striatal and spinal dopamine and its metabolite levels in control and MPTP-treated monkeys. Mean (% of control ± SD) regional DA, HVA and DOPAC levels were obtained from 3 controls and 3 MPTP-treated animals. Note the significant drop in DA and DOPAC levels in the striatum following MPTP intoxication. In the lumbar spinal cord, note the absence of DA level modification, the significant decrease in metabolite levels and the DA turnover index (ratio (HVA+DOPAC)/DA) in MPTP-treated animals compared to controls (*P<0.05; **P<0.001; ***P<0.0001, two-tailed *P* value, unpaired *t*-test). Abbreviations: DA = Dopamine, DOPAC = 3,4-Dihydroxyphenylacetic acid, HVA = Homovanillic acid.

As a significant cell loss was found in the A11 group following MPTP intoxication, we determined the DA and metabolite concentrations in the striatum and lumbar spinal cord (Fig. 10). As expected from previous work [32], [35], a significant decrease in DA and DOPAC concentrations was observed in the striatum of MPTP-treated animals compared to controls (respectively *P* = 0.026 and *P*<0.0001; unpaired *t*-test). Moreover, the DA turnover index, i.e. the ratio of DA metabolites (DOPAC + HVA) divided by the concentration of DA, which reflects the relation between DA metabolism and DA release, showed a significant increase in the striatum compared to control animals (P = 0.032; unpaired *t*-test). In spinal cord, HPLC dosages did not show any significant changes in DA concentrations following MPTP intoxication compared to control animals (*P>0.05*; unpaired *t*-test). However, the levels of the DA metabolites HVA and DOPAC were significantly lowered compared to those in control animals (respectively *P* = 0.0038 and *P* = 0.048; unpaired *t*-test). Consequently, the DA turnover index was significantly decreased (P = 0.011; unpaired *t*-test), which is indicative of an ongoing compensatory mechanism [46], [47].

## Discussion

In this study, we characterize for the first time the neuroanatomical aspect of the diencephalospinal pathway in the NHP and demonstrate the remarkable anatomical correspondence between the distribution of human and NHP hypothalamic TH-IR neurons. The A11 group is the unique TH-IR cell group projecting to the spinal cord with some fundamental differences compared to other dopaminergic systems, notably regarding the absence of DAT and AADC co-expression. The site(s) of action of the final end-product of these monoenzymatic TH-IR neurons, L-DOPA, remains to be determined. The demonstration, however, of AADC expressing neurons in the spinal cord supports the hypothesis of a local dopaminergic synthesis. As D1 is not expressed in the NHP spinal cord, dopamine could modulate the sensorimotor processes mainly through the D2 and D3 receptors.

### Topographic distribution of dopaminergic receptors within the lumbar spinal cord

The lack of D1 expression is a very relevant finding since it highlights inter-species differences notably with rodents in which D1 receptors are highly expressed in the spinal ventral horn [48], [49], [50]. Indeed, in rodents, evidence of a D1-mediated dopaminergic effect on locomotor spinal networks activation was shown. Dopamine or D1/D5 agonists administration was found to acutely elicit fictive locomotor activity in neonatal mice [51], [52] and rhythmic locomotor-like movements, both in wild type and in D5-KO spinal cord-transected mice [53], [54]. Also, in reduced models using isolated rat newborn spinal cord, high DA or D1 agonist concentration was shown to activate the central pattern generators [9], [55]. On the other hand, activation of spinal D1 receptor subtype was also implicated in pain modulation by increasing the long term potentiation of C-fibers or by stimulating the substance P and calcitonin release in rat spinal dorsal horn [56], [57]. In our study, the absence of the major activating dopaminergic receptors subtype D1 therefore question the so far considered predominant role of D1 receptor in locomotion and pain control. Further physiological studies in NHP spinal cord are needed to definitively address this issue.

Both the lack of D1 receptors in the NHP spinal cord and the striking correspondence between the anatomy of the NHP and human central nervous system underscore the need to extend findings in rodents to larger animals before inferring results obtained in lower species to humans. By contrast, we show that the D2 and D3 receptor subtypes are expressed in the lumbar spinal cord as in the rat where the D2 [58], [59] and D3 [60] receptor subtypes are highly expressed. Activating these two receptors leads to the inhibition of cAMP formation [61] and to a likely decrease in the neuronal activity of the expressing neurons [38]. In spinal cord, an inhibitory role of DA has also been suggested [62], [63]. As D2 receptors are more highly expressed in the dorsal horn, their stimulation might contribute to modulating the somesthetic processes and spinal reflexes [13]. The much wider distribution of D3 receptors suggests an extended role for these receptors in modulating sensorimotor integration. Identifying the phenotype of spinal cells expressing the various DA receptors is critical for better understanding of the DA modulation of the spinal cord functions.

### Anatomical organization of the diencephalospinal pathway

The rat diencephalospinal system was considered dopaminergic because of TH neuronal expression [5], [6], [64]. We now provide evidence in NHP that it originates almost exclusively from a caudal hypothalamic TH-IR cell group referred to as A11 [65]. Most of the A11-FG-labeled neurons were located ipsilaterally but a small proportion of the FG-labeled neurons was also found contralaterally. Our obervation is in line with Skagerberg et al. who demonstrated that A11 projections are predominantly ipsilateral in the rat [6]. Despite our efforts to confine FG injections to only one side of the cord, an unavoidable diffusion of FG was noticed on the contralateral side that directly could account for the 8% of TH-IR neurons labeled retrogradely on the contralateral A11 region. Decussation at the spinal or supra-spinal level cannot be ruled out but would be modest, if any. Indeed, if there were a few contralateral axonal projections, the FG labeling could well be below detection threshold of our method.

Despite the assumed exclusive supra-spinal origin of spinal DA [39], [66], interspecies similarities and differences should be highlighted regarding the location of the TH-IR cell groups projecting to the cord. In our study, the retrograde tracer reached almost exclusively the TH-IR neurons of the A11 group, in keeping with rat data [6], [67]. This contrasts however with the results obtained in rabbit and mouse in which only the A13 group in the former [68] and all three A10, A11 and A13 groups in the latter [69] were shown to project to the spinal cord. Importantly, our results fit with the single anatomical human description of A11 and beyond, with the entire human hypothalamic TH-IR regions [40]. This remarkable anatomical correspondence between the NHP and human further supports the relevance of NHP for studying the physiology of the hypothalamic DA circuitry and the downstream functional consequences of its dysfunction. In the same way, at the spinal cord level, the predominant localization of D2 receptors in the dorsal horn confirms earlier investigations in the rat [67] and perfectly matches the high dopaminergic fiber density in the dorsal horn of both rodents and NHP spinal cord [39].

### Particular phenotype of the A11 neurons: towards an L-DOPA diencephalospinal system?

The phenotypic characterization of A11 neurons showed that, although they are TH-immunopositive, they do not express DBH or AADC. This particular phenotype therefore questions the validity of the so far unquestioned dopaminergic nomenclature of this area. Monoenzymatic TH-positive neurons have been mainly described in the rat and the NHP arcuate nucleus [70], [71], [72], [73], [74] and, one other study using antibodies against L-DOPA and dopamine in the rat posterior hypothalamus also described a majority of L-DOPA-containing neurons within the A11 region [75]. Here we show for the first time the monoenzymatic TH nature of the neurons in the NHP A11 group. Thus, L-DOPA synthesis in these neurons as a final releasable product raises questions about their functional significance. Although it has been suggested that L-DOPA plays a role in locomotion processes in cats and rodents [76], [77], it remains unclear whether this medullar action is due to L-DOPA per se or to its conversion to DA or noradrenaline. As we also demonstrate the presence of monoenzymatic AADC neurons in the NHP spinal cord and because DA could be synthesized by non-dopaminergic neurons [78], [79], we speculate that part of the spinally released L-DOPA may be locally converted into dopamine. Otherwise, the contiguity between the TH-IR and AADC-IR neurons in the posterior hypothalamus might imply a cooperative synthesis of DA, as already suggested in the rat arcuate nucleus [80]. However, it cannot be ruled out that these neurons express AADC but at levels too low to be detected by immunohistochemistry, especially since the levels of AADC expression seemed variable [81] and may vary according to the circadian rhythm. In the same way, dopamine immunopositives fibers were found in NHP spinal cord [39] with a distribution consistent with the A11 labeling previously observed in rats [67]. Regarding our finding of a L-DOPAergic nature of A11 neurons, this discrepancy requires further investigations, but one may argue in favor of a possible AADC/TH colabeling in spinal cord descending collaterals.

Finally, A11 neurons do not express DAT. This is consistent with previous reports in rats and human in which a lack of DAT was found in the hypothalamus [82], [83]. The absence of DAT provides additional evidence against the so far presumed DA nature of A11 neurons.

### Consequences of MPTP administration on A11 diencephalospinal pathway

The active metabolite of MPTP, i.e. MPP+, requires the DAT to be uptaken into dopaminergic neurons to exert its toxicity [84], [85]. Here, despite the lack of DAT expression in A11 neurons, we found a significant cell death in this area following MPTP intoxication. However, the MPTP toxic effect is not limited to the DA regions and affects other monoaminergic [86] or non-monoaminergic regions [87], [88] that do not express DAT either. As our intoxication paradigm used high MPTP doses and given that MPTP is highly lipophilic [89], a passive uptake of MPTP in A11 neurons combined with an absence of the neuroprotective protein calbindin 28 k may explain the partial cell loss. Our data are in line with previous findings showing that different TH-IR cell groups are not uniformly affected by MPTP [32], [36]. Indeed, the 50% MPTP-induced cell loss in A11 is lower than the 90% and 70% loss reported in the A9-10 and A8 groups respectively, which both express DAT.

The A11 cell loss in MPTP-treated monkeys did not induce significant changes in spinal DA concentration. However, spinal DA metabolite concentrations were significantly reduced. As MPTP induced a partial lesion of A11 neurons, a compensatory downregulation of DA spinal catabolism may be sufficient to maintain a normal basal spinal DA tone [47]. Nevertheless, fine measurements of spinal DA concentration following MPTP intoxication by voltametry could be useful for assessing the potential impairment of DA transmission, notably when the diencephalospinal system is activated.

### Potential relevant pathophysiological implications

Spinal cord excitability impairment may manifest as RLS sensorimotor symptoms, a disorder characterized by an urge to move the limbs and unpleasant sensations in the legs occurring at rest and particularly in the evening [90]. These symptoms are partially and temporarily relieved by movement and are particularly responsive to dopaminergic agents, thus supporting the hypothesis of an involvement of the diencephalospinal pathway [10], [21]. Clinical and electrophysiological studies in patients with RLS have indeed provided evidence of a sensorimotor processing impairment, suggesting an enhanced spinal cord excitability and/or supraspinal pain modulation that is reversed by dopaminergic treatment [91], [92]. In hyperactive D3-receptor knock out mice [93], it was also shown that D3 spinal receptors are involved in limiting the spinal cord excitability [12] thus bringing more fundamental evidence consistent with an involvement of spinal dopamine dysfunction in the etiology of RLS. As DA spinal release increases with locomotion [18], [19], the relief of RLS symptoms by movements could be partly explained by a comparable mechanism. Similarly, the implication of A11 in chronic pain conditions such as migraine has also been demonstrated [11]. Migraine is more prevalent in patients with RLS [94], [95], [96], suggesting, at least in part, a common pathophysiological mechanism involving the diencephalospinal pathway.

In animals, lesioning of the A11 group has been attempted in rodents in order to model RLS [49], [97] and to investigate the dopaminergic influences in migraine [11]. In the first set of studies, however, the clinical phenotype resulting from such damage could hardly be considered as RLS symptoms. Large animals might offer a wider clinical directory and our work constitutes the first step toward the development of models targeting the A11 group in the NHP.

In conclusion, a growing body of evidence supports the hypothesis of a hypothalamic involvement in the physiology of the RLS and that of the A11 area in the control of nociception processes [11], [98]. By reinstating a clearer anatomical description of the diencephalospinal pathway in the NHP, we believe that our results will contribute to better understanding of the physiology of this tract and the ensuing consequences of its dysfunction.
